# Prognostic Value of Dynamic Monitoring of Platelet Counts and Red Cell Distribution Width to Platelet Ratio in Patients with Acute Type A Aortic Dissection

**DOI:** 10.21470/1678-9741-2020-0132

**Published:** 2020

**Authors:** Jianxun Zhao, Dongze Li, Hong Liu

**Affiliations:** 1 Department of Cardiology, West China Hospital, Sichuan University, Chengdu, China.; 2 Department of Emergency Medicine, Laboratory of Emergency Medicine, West China Hospital, Sichuan University, Chengdu, China.

**Dear editor,**

We read with great interest the article by Bedel et al.^[1]^ regarding the association of neutrophil-to-lymphocyte ratio (NLR) and platelet-to-lymphocyte ratio (PLR) with in-hospital mortality in patients with acute type A aortic dissection (AAAD). The authors included 96 AAAD patients and indicated that NLR and PLR were independent risk factors for in-hospital mortality of AAAD patients^[1]^. AAAD is a cardiovascular emergency with high pre-hospital and perioperative mortality, despite the progress of diagnosis and management^[2].^ It is important for patients with AAAD to determine the risk factors associated with the prognosis as soon as possible. It was widely accepted that immune-inflammatory mechanisms contribute to aortic rupture and remodeling^[3]^. Previous studies have shown that C-reactive protein (CRP) and platelet count (PLC) were associated with the prognosis in AAAD patients^[4]^. However, the significance of dynamic monitoring of inflammation markers in the prognosis of patients with AAAD is still unknown. Here, we report four AAAD patients to attempt to explore the prognostic value of dynamic monitoring of inflammation markers in patients with AAAD.

**Fig. 1:**
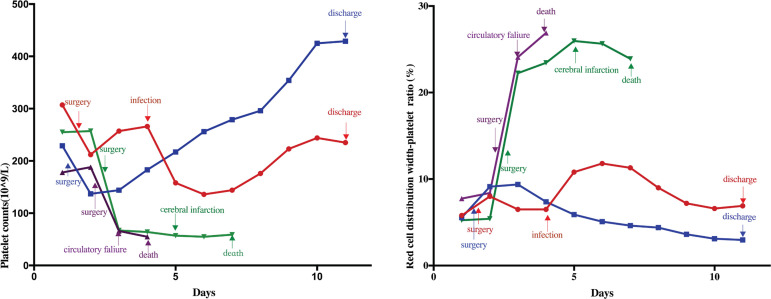
Dynamic variation of platelet counts (A) and red cell distribution width-platelet ratio (B) (red line - case 1, blue line - case 2, violet line - case 3, green line - case 4).

Patient 1. A 56-year-old man with a history of hypertension was admitted to the chest pain center of emergency department with a sudden onset of 10-hour substernal chest pain radiating to the back and was diagnosed with AAAD by contrast-enhanced multidetector computed tomography (CT). It originated in the root of the aorta and extended distally to the origin of the left common iliac artery. An emergency total arch replacement and stented elephant trunk procedure was performed. PLC decreased from 307×109/L to 212×109/L, along with an increase in red cell distribution width to platelet ratio (RPR) (5.8% to 8.0%) (Table 1 and Figure 1B). Two days after the operation, the patient suffered from pneumonia and PLC decreased from 266×109/L to 144×109/L. RPR increased from 6.5% to 11.3% after the pneumonia. As the condition improved, PLC increased to 244×109/L and RPR dipped to 6.6%. The patient was discharged on day 11.

**Table 1:** Hematological parameters of the cases.

	Day										
	1	2	3	4	5	6	7	8	9	10	11
**Case 1**											
WBC (109/L)	6.9	11.6	8.2	18.2	18.5	17.4	17.1	17.9	18.0	18.0	14.3
Neu (%)	76.4	95.4	94.7	96.6	86.3	84.2	86.9	86.8	80.0	88.3	86.4
Lym (%)	12.8	1.7	2.7	1.6	6.9	7.0	6.4	6.6	8.0	6.0	6.1
Hb (g/dL)	177	130	122	145	171	164	151	157	163	149	147
RDW (%)	17.9	16.9	16.7	17.4	17.0	16.1	16.2	15.9	16.0	16.0	16.3
PLC (109/L)	307	212	257	266	158	136	144	176	223	244	235
RPR (%)	5.8	8.0	6.5	6.5	10.8	11.8	11.3	9.0	7.2	6.6	6.9
**Case 2**											
WBC (109/L)	15.0	6.1	8.4	11.2	12.1	11.56	13.1	10.8	11.0	12.7	7.5
Neu (%)	91.4	87.1	87.1	84.7	80.1	80.0	83.5	83.2	83	84.2	82.6
Lym (%)	4.1	9.4	3.6	6.6	7.3	8.0	4.6	5.0	6.1	6.0	7.6
Hb (g/dL)	153	95	104	101	128	133	124	114	122	110	103
RDW (%)	12.7	12.5	13.5	13.5	12.8	13.0	12.9	13.0	12.8	13.2	12.7
PLC (10^9^/L)	229	137	144	183	217	256	279	296	354	425	429
RPR (%)	5.6	9.1	9.4	7.4	5.9	5.1	4.6	4.4	3.6	3.1	3.0
**Case 3**											
WBC (109/L)	9.0	8.5	8.7	14.3							
Neu (%)	87.3	85.1	77.0	83.6							
Lym (%)	8.3	11.1	15.3	10.2							
Hb (g/dL)	121	93	101	89							
RDW (%)	13.8	15.8	15.9	14.8							
PLC (109/L)	178	188	66	61							
RPR (%)	7.8	8.4	24.1	24.3							
**Case 4**											
WBC (109/L)	11.9	13.6	18.7	15.8	10.8	15.4	19.3				
Neu (%)	89.8	88.9	93.5	88.8	89.8	76.0	82.6				
Lym (%)	9.4	9.5	3.0	3.9	5.5	10.7	7.6				
Hb (g/dL)	156	153	110	92	111	117	114				
RDW (%)	13.4	13.9	14.9	15.0	14.8	14.1	14.1				
PLC (10^9^/L)	255	257	67	64	57	55	59				
RPR (%)	5.3	5.4	22.2	23.4	26.0	25.6	23.9				

Reference ranges: white blood cells (WBC)=3.5-9.5×10^9^/L; neutrophils proportion (Neu)=40-75%; lymphocytes proportion (Lym)=20-50%; hemoglobin (Hb)=115-150×10^9^/L; red cell distribution width (RDW)=11.5-14.5%; platelet count (PLC)=100-300×10^9^/L

Patient 2. A 38-year-old previously healthy man was admitted after 10 hours of sudden chest pain. A contrast-enhanced multidetector CT showed an aortic dissection of the aortic root to the left iliac artery and emergency surgery was performed. The day after operation, PLC decreased from 229×109/L to 137×109/L, and had a slow increase before reaching a plateau (425×109/L and 429×109/L) on days 10 and 11 (Table 1 and Figure 1A). RPR raised from 5.6% to 9.1% after the operation and slowly declined to 3.0% when the patient was discharged uneventfully on day 11 (Table 1 and Figure 1B).

Patient 3. A 61-year-old woman with a history of hypertension was admitted with a severe chest pain for 15 hours. The CT scan demonstrated evidence of dissection originating from the aortic arch, extending to the abdominal aorta. The patient underwent an ascending aortic graft and hemi-arch replacement on day 2. PLC decreased from 178×109/L to 66×109/L and RPR increased from 7.8% to 24.1% after operation (Table 1 and Figure 1B). The patient suffered a circulatory collapse after the surgery and died on day 4.

Patient 4. A 70-year-old man with a history of hypertension presented to the emergency department with chest pain for 8 hours. CT showed an AAAD originating from the ascending aorta and extending to the proximal aortic arch. The patient underwent surgery on day 2. PLC decreased from 255×109/L to 67×109/L and RPR increased from 5.3% to 22.2% (Table 1 and Figure 1B). The patient became unconscious on the 4^th^ postoperative day, and the head CT scan revealed cerebral infarction. PLC was consistently low (55×109/L to 59×109/L) and RPR remained at a high level (25.6% to 23.9%). The patient died of respiratory and circulatory failure on the 6^th^ postoperative day.

Generally, the PLC in AAAD patients is decreased during surgery and on the 1^st^ postoperative day, due to the consumption in surgery and perioperative bleeding^[5]^. Along with artery repair, bone marrow activity and decreased inflammatory response, the PLC would increase. However, AAAD is characterized by systemic inflammatory response syndrome (SIRS), and postoperative complications, such as pneumonia and circulatory failure, would significantly increase the systemic inflammatory and anti-inflammatory responses. Thus, PLC would decrease continuously or remain at a low level. In this study, decreases of PLCs were observed among the four patients on the 1^st^ postoperative day. Thereafter, the PLC of patient 2 quickly returned until he was discharged. The PLC of patient 1 also returned after surgery, but then decreased because of infection. After the infection was well controlled, the PLC continuously increased until he was discharged. However, for patients 3 and 4, the PLC continuously decreased because of circulatory failure, and they died within a few days.

Besides, red cell distribution width (RDW) and RPR also played a critical role in inflammation and immune responses. Sbarouni et al.^[6]^ reported that RDW and RPR were significantly higher in patients with AAAD compared to aortic aneurysms and normal subjects, but not associated with mortality. In this study, the RDW levels changed slightly among the four patients until they died or were discharged, and the RPR levels were comparable among the four patients on admission. However, sharp increases in RPR were observed in patients 3 and 4 until they died. However, for patients 1 and 2, the RPR levels just changed slightly until they were discharged.

Taken together, these results indicated that dynamic monitoring of PLC and RPR is important for patients with AAAD and might have predictive value for prognosis in patients with AAAD.

The strength of this study is that it is the first attempt to explore the prognostic value of dynamic monitoring of inflammation markers in patients with AAAD. The limitation of this study is the small number of patients. Even so, this study sheds lights on the importance of dynamic monitoring of inflammation markers in patients with AAAD. Further studies with larger number of patients are needed to verify our results.
